# MRI with gadofosveset: A potential marker for permeability in myocardial infarction

**DOI:** 10.1016/j.atherosclerosis.2018.04.024

**Published:** 2018-08

**Authors:** Begoña Lavin, Andrea Protti, Silvia Lorrio, Xuebin Dong, Alkystis Phinikaridou, René M. Botnar, Ajay Shah

**Affiliations:** a School of Biomedical Engineering Imaging Sciences, King's College London, London, UK; b The British Heart Foundation Centre of Excellence, Cardiovascular Division, King's College London, London, United Kingdom; c Cardiovascular Division, James Black Centre, King's College Hospital Denmark Hill London, London, SE5 9NU, United Kingdom; d Pontificia Universidad Católica de Chile, Escuela de Ingeniería, Santiago, Chile

**Keywords:** Magnetic resonance imaging, Myocardial infarction, Albumin, Permeability, Remodeling

## Abstract

**Background and aims:**

Acute ischemia is associated with myocardial endothelial damage and microvessel formation, resulting in leakage of plasma albumin into the myocardial extravascular space. In this study, we tested whether an albumin-binding intravascular contrast agent (gadofosveset) allows for improved quantification of myocardial permeability compared to the conventional extracellular contrast agent Gd-DTPA using late gadolinium enhancement (LGE) and T1 mapping *in vivo*.

**Methods:**

MI was induced in C57BL/6 mice (n = 6) and cardiac magnetic resonance imaging (CMR) was performed at 3, 10 and 21 days post-MI using Gd-DTPA and 24 h later using gadofosveset. Functional, LGE and T1 mapping protocols were performed 45 min post-injection of the contrast agent.

**Results:**

LGE images showed that both contrast agents provided similar measurements of infarct area at all time points following MI. Importantly, the myocardial R_1_ measurements after administration of gadofosveset were higher in the acute phase-day 3 (R1 [s^−1^] = 6.29 ± 0.29) compared to the maturation phase-days 10 and 21 (R1 [s^−1^] = 4.76 ± 0.30 and 4.48 ± 0.14), suggesting that the uptake of this agent could be used to stage myocardial remodeling. No differences in myocardial R1 were observed after administration of Gd-DTPA at different time points post-MI (R1 [s^−1^] = 3d: 3.77 ± 0.37; 10d: 2.74 ± 0.06; 21d: 3.35 ± 0.26). The MRI results were validated by *ex vivo* histology that showed albumin leakage in the myocardium in the acute phase and microvessel formation at later stages.

**Conclusions:**

We demonstrate the merits of an albumin-binding contrast agent for monitoring changes in myocardial permeability between acute ischemia and chronic post-MI myocardial remodeling.

## Highlights

• *In-vivo* measurements of LGE areas using gadofosveset after MI are comparable to the gold-standard, Gd-DTPA.
• *In-vivo* imaging using gadofosveset shows higher signal intensity making delineation of the ischemic myocardium easier.
• T1 mapping using gadofosveset allows monitoring changes in myocardial permeability.
• T1 mapping using gadofosveset allows differentiation between acute and chronic phases post-MI, compared to Gd-DTPA.

## 1 Introduction

Myocardial infarction (MI) remains the leading cause of heart failure, morbidity and mortality in the Western societies [1]. Post-MI remodeling is generally thought to be subdivided in two successive and overlapping phases: an acute inflammatory stage and a chronic maturation stage [2]. The first is characterized by the influx of leukocytes (neutrophils and inflammatory monocytes) to release inflammatory mediators and remove cellular debris [3] while the second is initiated by the influx of reparative monocytes that orchestrate the healing response which includes the deposition of collagen and elastin and the formation of microvessels to restore blood supply [4]. These processes are regulated by a complex signaling cascade leading to transcriptional, structural, electrophysiological, and functional events occurring within the cardiomyocytes [5]. Remodeling is therefore a dynamic and time-dependent process, with changes occurring in both the necrotic region and the adjacent non-infarcted remote myocardium [6,7].

The extent of myocardial damage and its location within the left ventricle (LV) directly affects the magnitude of LV remodeling [8]. The underlying mechanisms of LV remodeling are closely related to the infarction itself, including cell death and loss of contractile activity within the affected zone and secondary ventricular dilation and remodeling in the LV regions remote to the infarct as a result of increased hemodynamic burden [9]. It is well established that the endothelium is significantly damaged during the acute stage of MI [10,11] and associated with an increase in the intercellular junction width [12,13]. Normal junctions allow the transport of small water-soluble molecules up to a diameter of 2 nm [14] whereas breaks in the tight junctions allows for the transport of molecules up to 20 nm diameter and more [15,16] facilitating the influx of serum albumin (diameter of ≈6 nm). At the late stage of MI, gap junction width seems to reverse to normal size based on electron microscopy studies [17], thereby decreasing leakage of large molecules into the diseased tissue [13].

Animal models of MI are important in research to understand the complex pathophysiology of ischemic heart disease [18] and are essential for testing therapeutic approaches for the treatment of MI. There are currently two widely used murine models of left anterior descending (LAD) ligation to induce myocardial infarction: a permanent ligation of the LAD, as used in this study, and an ischemia reperfusion injury model. Similar to human MI, interruption of blood flow to the myocardial territory supplied by the LAD produces profound ischemia in the anterolateral territory of the heart which then manifests as an acute MI. Both models have been extensively used for the better understanding of the underlying mechanisms of post-MI remodeling at the cellular and molecular levels [[19], [20], [21], [22]]. Briefly, permanent ligation of the LAD is associated with ischemic necrosis and increased inflammation, whereas the ischemia-reperfusion injury model is associated to cell death via apoptosis and limited ischemic necrosis [23]. Additionally, these models have been used to assess the changes in cardiac function at different time points following injury using different imaging modalities. Both models lead to cardiac dysfunction including systolic and diastolic dysfunction associated with adverse myocardial remodeling that could lead to the development of heart failure [[24], [25], [26]]. Albumin is the most abundant protein in human plasma, accounting for half of all serum proteins [27]. Approximately 33% of albumin can be found in the intravascular compartment, while the remaining 67% is in the extravascular exchangeable and remote compartments [28]. In diseases such as atherosclerosis [29] and myocardial infarction [30], an increase in albumin leakage is expected due to acute endothelial damage and later microvessel formation [31].

In order to investigate focal changes in myocardial permeability, which may provide a non-invasive tool for the assessment of ischemic endothelial damage in infarcted myocardium [32] and microvessel formation, we used cardiac magnetic resonance imaging (CMR). Late gadolinium enhancement (LGE) MRI is the gold-standard technique to estimate infarct area after injection of Gd-DTPA (gadolinium diethylene triamine pentaacetic acid) [[33], [34], [35]]. We hypothesize that injection of the intravascular albumin-binding contrast agent gadofosveset may provide a measure of infarct area using LGE (similar to conventional Gd-DTPA) but also unveils temporal changes in myocardial permeability in acute ischemia using T1 mapping. To quantify gadofosveset uptake as a non-invasive surrogate measure of permeability we performed T1 mapping of the myocardium in addition to high resolution LGE imaging for direct infarct visualization. Gadofosveset, commercially known as Ablavar^**®**^, is a clinically approved gadolinium-based blood pool contrast agent that reversibly binds to serum albumin, resulting in a prolonged vascular presence and a 5–10-fold increase in relaxivity (r1) [[36], [37], [38]]. Gadofosveset may enter the interstitium through leaky microvessels [39] and mechanically damaged endothelium as we previously demonstrated in a mouse model of atherosclerosis [[39], [40], [41], [42], [43]]. We sought to investigate whether contrast-enhanced MRI using gadofosveset could provide information on changes in myocardial permeability to differentiate between acute ischemia and chronic post-MI remodeling using LGE and T1 mapping *in vivo* at high field.

## 2 Materials and methods

### 2.1 Animal model

**Fig. 1:**
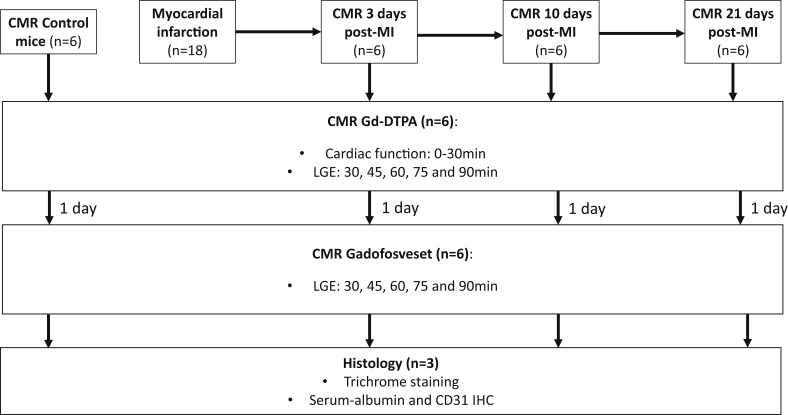
Experimental protocol, contrast agents used, timing of MRI scans and histological protocols performed.

In this longitudinal study, 6 female wild-type C57BL/6 mice weighing 18–24 g were purchased from Harlan Laboratories (Blackthorn, United Kingdom). Left coronary artery permanent ligation was used to induce myocardial infarction (MI). The protocol design is detailed in Fig. 1. Surgery was performed with 1.5% isoflurane and a mix of O_2_/medical air at a flow rate of 2 l/min. Animals underwent endotracheal intubation and were ventilated using a dedicated small animal ventilator (Hugo Sacks Elektronic, Germany). A lateral thoracotomy was made, the chest wall muscles were incised and reflected, and the thorax opened in the fourth intercostal space. The pericardium was removed to access the epicardial surface. The left coronary artery was ligated using 8/0 Ethilon suture, at a level between 1 and 2 mm below the tip of the left atrium. Successful ligation was confirmed by regional blanching of the left ventricle, extending to the apex. The chest wall was then repaired in layers and the animals weaned from the ventilator. Mice were recovered in a warmed chamber for at least 6 h. Perioperative analgesia with buprenorphine (0.15 mg/kg) intramuscularly and flunixin (2.5 mg/kg) subcutaneously was used. All procedures used in these studies were performed in accordance with institutional guidelines, following the European Communities Council Directive 2010/63/EU on the protection of animals used for scientific purposes, and UK Home Office legislation (The Animals (Scientific Procedures) Act 1986).

### 2.2 *In vivo* CMR at 7 T

*In vivo* CMR was performed in 6 mice using a 7 T horizontal MR scanner (Agilent, Varian Inc., Palo Alto, CA) equipped with a gradient coil with an inner diameter of 12 cm and gradient strength and rise-time were 1000 mT/m (100G/cm) and 120 μs, respectively. A quadrature transmit/receive coil (RAPID Biomedical GmbH, Germany) with an internal diameter of 39 mm was used. Six mice were imaged prior to and at 3, 10 and 21 days post-MI after intraperitoneal (i.p.) injection of 0.75 mmol/kg Gd-DTPA (Magnevist^®^, Bayer Schering Pharma AG, Berlin-Wedding, Germany). Mice were imaged in prone position at 30, 45, 60, 75 and 90 min after Gd-DTPA administration. After a washout period of 24 h, 0.75 mmol/kg of gadofosveset trisodium (Ablavar^®^, Lantheus Medical Imaging, North Billerica, MA) was administered i.p. in the same mice and CMR was repeated at 30, 45, 60, 75 and 90 min after Gadovosveset administration. Anaesthesia was induced with 5% and maintained with 1–2% isoflurane during the CMR session and the body temperature was maintained at 37 °C using warm air fan (SA Instruments, Stony Brook, NY). The electrocardiogram (ECG) was monitored using two metallic needles placed subcutaneously in the front paws. A pressure-transducer for respiratory gating was placed on the abdomen of the mice. Following scout scans, multi slice Cine-FLASH images were acquired to measure functional and volumetric parameters with a FOV = 25 × 25 mm^2^, slide thickness = 1 mm, matrix size 128 × 128, 9 to 10 frames/cycle, 9 slices, flip angle = 40°, cardiac cycle = 120 ± 30 ms, acquisition time ≈8 min. An ECG triggered, single slice, Look-Locker acquisition was used for T1 mapping and to measure R1 values of the remote and infarcted myocardium. The slice was selected based on the maximum infarct extension detected on cine-FLASH images. T1 mapping was also employed to obtain R1 values for pharmacokinetic measurements. LGE images were obtained from one frame of the T1 mapping sequence and used for the measurement of infarct area. Imaging parameters included FOV = 25 × 25 mm^2^, slice thickness = 1 mm, matrix size = 128 × 128, 3 phases/cycle, total of 30 phases, 1 slice, flip angle = 10°, TR = 2700 ms, TReff ≈40 ms ((cardiac cycle)/(3 phase/cycle)), TE = 2 ms, BW = 10 MHz, cardiac cycle = 120 ± 20 ms, number of averages = 1, acquisition time ≈ 13 min.

### 2.3 CMR data analysis

Functional and volumetric parameters were calculated from cine-FLASH images and areas of contrast-enhancement were calculated using LGE images with a semi-automated in-house developed computer software program (King's College London, ClinicalVolumes). Ejection fraction (EF), left ventricular end-diastolic volume (LVEDV), left ventricular end-systolic volume (LVESV), stroke volume (SV), and left ventricular (LV) mass were measured to evaluate the effect of MI on cardiac function and remodeling at all time points as previously reported [44].

Look-Locker T1 mapping resulted in 30 images (3 per cardiac cycle) from which R1 values of blood, infarcted, remote and healthy myocardium were calculated using an exponential 3 parameter fit (A-B*exp (-TI/T1*)) with subsequent T1 correction (OriginLab Corporation, Wellesley, USA). The inversion delays (TI) ranged from 120 ms to 1364 ms for a mouse with a heart rate of 400 bpm (RR = 120 ms). A flip angle correction was introduced to determine the T1 values as follows [45]:

[1] $$T_{1\;}=\;T_{1\;\;}^{*}(B/A-1)$$

For T1 mapping and LGE analysis, images were segmented at the end-diastolic phase due to the better contrast between the enhanced and remote areas compared to other time points during the cardiac cycle.

Contrast-to-noise ratio (CNR) between infarcted and remote myocardium was calculated using the following equation:

[2] $$CNR_{infarct/remote}=\frac{SI_{infarct}-SI_{remote}}{SD_{noise}}$$

No dedicated noise scan was used. Noise was measured in air, outside of the mouse. No Rayleigh correction was applied.

### 2.4 Histological analysis

At the end of the CMR scans, mice (n = 6) were culled by cervical dislocation and the hearts were harvested. An additional 3 hearts were used for histology at 3 and 10 days post-MI. Hearts were perfused with saline, harvested and immersed in 10% formalin for 48 h at 4 °C. Hearts were then embedded in paraffin and sectioned into 5 μm-thick transverse slices. Sections were stained with Masson's trichrome (Sigma-Aldrich, Dorset, UK) to assess tissue morphology and visualize the infarct, CD31 immunohistochemistry to detect endothelial cells and microvessels, and albumin immunohistochemistry for intraventricular albumin detection.

For CD31 immunohistochemistry, sections were immersed in 3% H_2_O_2_ in methanol to block endogenous peroxidase. Sections were then immersed in 0.01 M citrate buffer, pH 6.0, and boiled for 3 min, washed and blocked for 1 h with 10% donkey serum and then incubated overnight with the primary antibody [rabbit anti-mouse CD31 (1:50; Abcam, Cambridge, UK)]. Sections were washed and incubated with anti-rabbit HRP Polymer [X-Cell Plus Universal Polymer HRP detection kit (Biocare LLC, Concord, CA)] followed by peroxidase substrate to detect the signal (Vector^**®**^ SG Peroxidase substrate; Vector Laboratories, Burlingame, CA). Sections were counterstained with nuclear fast red. For albumin immunohistochemistry, the same protocol was performed and samples were incubated overnight with the primary antibody [goat anti-mouse serum albumin (1:5000; Abcam, Cambridge, UK)]. Sections were washed and incubated with secondary antibody [donkey anti-goat biotinylated antibody (1:200; Abcam, Cambridge, UK)] followed by streptavidin-peroxidase complex to amplify the signal (ABC Vectastain^**®**^ kit; Vector Laboratories, Peterborough, UK), and diaminobenzidine/H_2_O_2_ solution to detect the signal (DAB peroxidase substrate kit; Vector Laboratories, Burlingame, CA). Sections were counterstained with hematoxylin. Negative control sections were incubated without the primary antibody.

### 2.5 Statistical analysis

Results are expressed as mean ± SEM. Statistical differences were determined using GraphPad Prism 5.0 (GraphPad software, Inc., La Jolla, California, USA). Differences between time points and contrast agents were analyzed using 1 way ANOVA followed by a Dunns *post hoc* multiple comparison test. Bland-Altman method was used to test the agreement between the two contrast agents. *P-values* < 0.05 were used to define statistical significance.

## 3 Results

### 3.1 Cardiac functional and volumetric parameters post-MI

**Fig. 2:**
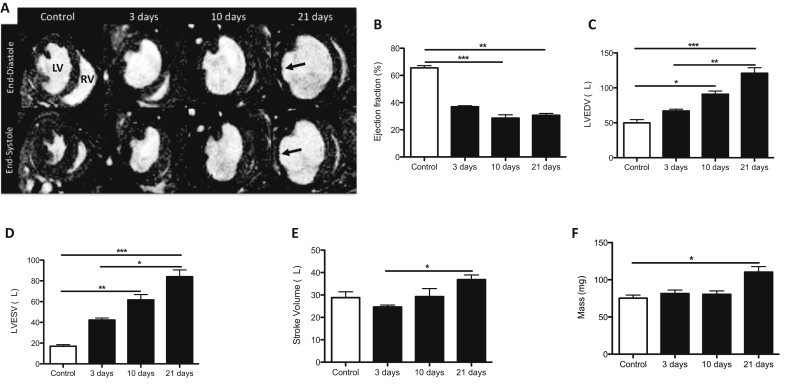
Cardiac function and volumetric parameters are altered after MI. (A) Representative end-diastolic and end-systolic short axis magnetic resonance images prior to and at 3, 10 and 21 days post-MI show progressive dilation of the LV and thinning of the LV wall (arrows). (B) Ejection fraction, (C) left ventricle end-diastolic volume (LVEDV), (D) left ventricle end-systolic volume (LVESV), (E) stroke volume and (F) mass show alteration of all functional and volumetric parameters after MI (n = 7). Significant functional and volumetric differences are reported by asterisks (*p < 0.05, **p < 0.01, *p* < 0.001).

Cine-FLASH images were used to assess ejection fraction, left ventricular (LV) volumes and mass (n = 6). Representative diastolic and systolic short-axis images of control, 3, 10 and 21 days post-MI hearts showed a significant enlargement of the LV and myocardial wall thinning at the later stages of infarction (Fig. 2A; arrows). Functional and volumetric analysis revealed a decrease in the contractile function of the heart measured as ejection fraction (%EF) from 10 days post-MI onwards (Fig. 2B). A continuous increase in LV end-diastolic volume (LVEDV) and LV end-systolic volume (LVESV) were detected over time, corroborating the enlargement of the LV (Fig. 2C and D, respectively) and an increase of stroke volume and mass of the heart 21 days after MI (Fig. 2E and F).

### 3.2 Pharmacokinetics of Gd-DTPA and gadofosveset at 3 days post-MI

**Fig. 3:**
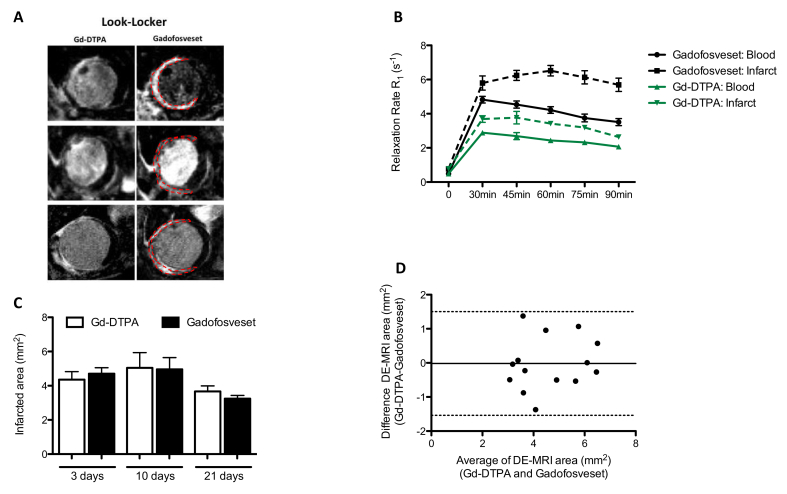
Pharmacokinetics and infarct area comparison between Gd-DTPA and gadofosveset. (A) Representative late gadolinium enhancement (LGE) images. The first column shows Gd-DTPA contrast uptake and the second column shows gadofosveset contrast uptake in the infarcted area at different time points post-MI. (B) Time-dependent changes in the relaxation rate (R_1_) of blood and infarcted tissue. The higher difference between blood and infarcted tissue occurred at 45 min after contrast administration with both contrast agents (n = 6). (C) Quantification of the contrast-enhanced infarcted areas measured with Gd-DTPA and gadofosveset (n = 6) shows good agreement, as corroborated by the corresponding Bland-Altman plot (D) where black line represents Gd-DTPA = gadofosveset; dotted black lines, upper and lower 95% limit of agreement between −1.54 and 1.50.

Pharmacokinetics of Gd-DTPA and gadofosveset (n = 6) were investigated at 3 days post-MI to determine the optimum imaging time point for MI imaging. The relaxation rate (R_1_) of both the ischemic myocardium and blood peaked at 30 min and was maintained for at least 90 min after administration of either Gd-DTPA or gadofosveset. However, gadofosveset showed increased R_1_ values compared to Gd-DTPA in the ischemic myocardium and blood (Fig. 3A and B) owing to its higher relaxivity upon binding to albumin. Based on the pharmacokinetic curves, subsequent CMR scans were performed at 45 min after injection of either contrast agent due to the bigger difference between ischemic myocardium and blood at this time point (Fig. 3B).

### 3.3 LGE assessment of ischemic area using Gd-DTPA and gadofosveset

Infarct area was similar as measured by LGE MRI after administration of either Gd-DTPA or gadofosveset (Fig. 3C). Sharper images and therefore easier delineation of the ischemic myocardium were achieved using gadofosveset at 3 days post-MI compared to corresponding Gd-DTPA images. However, at later time points the delineation of the ischemic area became more challenging due to the thinning of the ventricular wall irrespective of which contrast agent was used (Fig. 3A and C). Contrast-enhanced ischemic areas were similar at all time points post-MI as measured with both contrast agents (Fig. 3C). The agreement between the delayed-enhanced MRI area measured with Gd-DTPA or gadofosveset was also confirmed by using the Bland-Altman analysis (Fig. 3D), suggesting a role of gadofosveset as an alternative to Gd-DTPA for the assessment of area at risk.

### 3.4 *In-vivo* quantification of contrast agent uptake during MI remodeling using T1 mapping after Gd-DTPA and gadofosveset administration

**Fig. 4:**
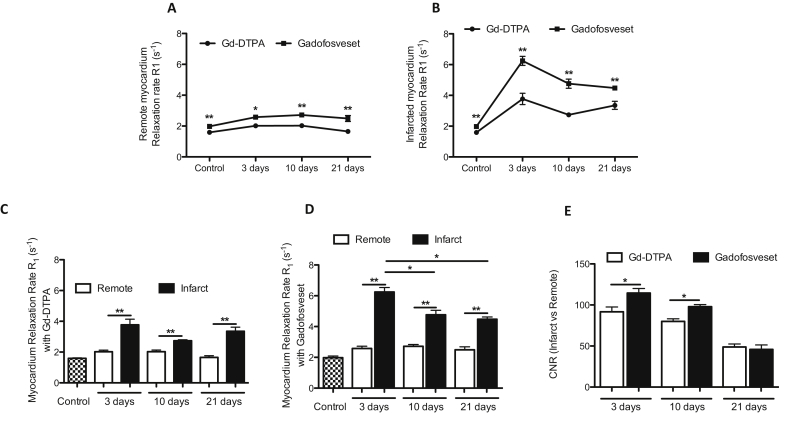
Acute endothelial damage and late microvessel formation can be measured using gadofosveset in the infarcted area over time. Increased R_1_ values are detected in the remote (A) and infarcted (B) myocardium over time when using gadofosveset compared to Gd-DTPA (n = 6). Significant differences are detected between remote and infarcted myocardium when using Gd-DTPA (C) and gadofosveset (D) (n = 6). In addition, gadofosveset uptake shows significant differences between time points post-MI differentiating between acute endothelial damage at 3 days and microvessel formation at later stages (D). (E) Increased contrast-to-noise ratio (CNR) is detected using gadofosveset at 3 and 10 days post-MI (n = 5).

Measurement of the relaxation rate (R_1_) *in situ* allows quantification of gadolinium uptake in the ischemic and remote myocardium. The analysis showed significantly higher R_1_ values in both the remote (Fig. 4A) and ischemic myocardium (Fig. 4B) after injection of gadofosveset compared to Gd-DTPA at all time points post-MI. The R_1_ values of the ischemic myocardium were higher compared to the remote myocardium after administration of both Gd-DTPA and gadofosveset at all time points post-MI (Fig. 4C and D). However, the R1 values of the ischemic myocardium showed significant differences between the acute phase (3 days post-MI) and the maturation phase (10 and 21 days post-MI) only after administration of gadofosveset (Fig. 4D). Finally, increased contrast-to-noise ratio (CNR) between remote and ischemic myocardium was observed at 3 and 10 days post-MI after injection of gadofosveset compared with Gd-DTPA. No differences were detected at 21 days (Fig. 4E). These results suggest that R_1_ measurements are a more sensitive method compared to CNR and can provide additional information about the evolution of MI.

### 3.5 *Ex vivo* histological findings

**Fig. 5:**
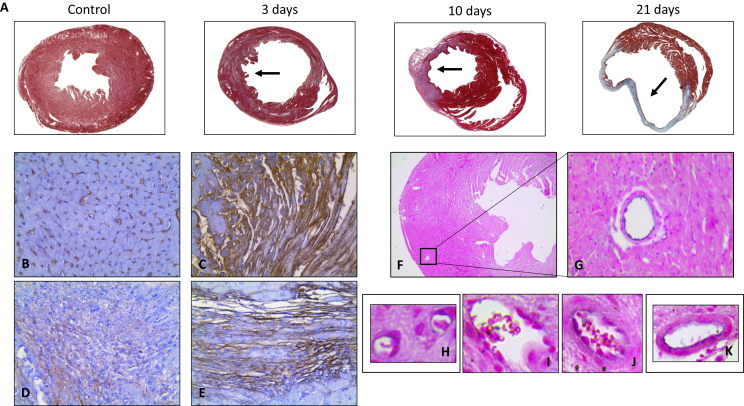
Increase albumin extravasation correlates with microvessel formation in the infarcted area over time. (A) Representative trichrome images prior to and at 3, 10 and 21 days post-MI where the infarcted area can be identified (arrows). Visual assessment of serum albumin immunohistochemistry (brown signal) suggested higher levels of albumin in the infarcted area 3 days after MI (C), when compared to the control (B). Images at 10 (D) and 21 (E) days post-MI show that albumin distribution is located mainly in the infarcted area. (F and G) Representative CD31 immunohistochemistry (black signal) in healthy myocardium. (H–K) Representative images of microvessels with different size and maturation state formed in the ischemic area 21 days post-MI.

*Ex vivo* histological analysis corroborated the *in vivo* MRI findings. Representative trichrome staining of hearts prior to and 3, 10 and 21 days post-MI showed the morphological changes (arrows) after MI, which were primarily characterized by a significant increase in collagen deposition (blue) in the ischemic area by day 21 (Fig. 5A). Albumin immunohistochemistry (albumin: brown signal, nuclei: purple) was used to detect leakage of blood albumin into the myocardium. In control animals, low albumin leakage was detected (Fig. 5B). However, significant albumin immunopositive areas in and throughout the area at risk were detected at 3 days after MI (Fig. 5C), confirming the increased leakage of albumin in the acute stage after MI as seen by MRI. At 10 and 21 days post-MI, a decrease in albumin staining compared to 3 days was detected. However, albumin staining was higher than in the control group and mainly located in ischemic myocardium (Fig. 5D and Fig. E, respectively). The remote myocardium showed similar albumin staining as compared to control mice (data not shown). CD31 staining (CD31: black signal, nuclei: pink signal) allowed visualization of microvessels. A representative image of the control (Fig. 5F) showed low number of vessels (Fig. 5G). Increased density of microvessels with different sizes and maturation phases were detected in the ischemic myocardium at 21 days post-MI. Representative images are presented in Fig. 5H–K, suggesting that this increase in microvessels could contribute to the elevated myocardial permeability of the infarcted area at later stages following MI as seen by MRI.

## 4 Discussion

Noninvasive assessment of cardiac permeability after MI can provide important diagnostic information not only on the extent of the ischemia, but also on the extravasation and distribution of macromolecules within the injured myocardium allowing the staging of different phases post-MI. In this study, we demonstrate that [1] measurements of LGE areas using gadofosveset are comparable to the gold-standard, Gd-DTPA. Importantly, gadofosveset shows higher signal intensity which makes visualization and delineation of the ischemic area easier. [2] Increased permeability measured by T_1_ mapping shows increased R_1_ relaxation rates 3 days post-MI, due to the acute endothelial damage that is maintained at later time points of MI due to microvessel formation in the infarcted myocardium. *In vivo* MRI analysis was validated using *ex vivo* measurements of permeability and microvessel formation using albumin and CD31 immunohistochemistry, respectively. We proposed that uptake of gadofosveset into the ischemic area measured by T_1_ mapping may help to differentiate between acute ischemia and chronic remodeling post-MI.

In our study, all mice showed progressive LV dilation that was accompanied with a reduction in global contractile function (ejection fraction around 40%) after MI. In addition, stroke volume and mass were significantly increased at later time points suggesting alteration of the heart. These measurements are in good agreement with other studies that evaluated cardiac function in this animal model using different imaging modalities [46,47]. Different Gd-based contrast agents are routinely used in the clinic for the assessment of scar size and % transmurality in patients after MI [48,49]. LGE imaging is usually performed using the extracellular contrast agent Gd-DTPA. Several cases of nephrogenic systemic fibrosis (NSF) in patients with renal failure have been reported after the administration of Gd-DTPA [50]. As an alternative, cyclic chelates such as Gd-DOTA (e.g. Gadobutrol) that are classified as low-risk media and have higher relaxivity compared to Gd-DTPA (Magnevist): r1 = 3.9 mmol^−1^s^−1^; Gd-DOTA (Gadobutrol): r1 = 4.7 mmol^−1^s^−1^) at 1.5 T are currently used in the clinic [51]. Although these contrast agents provide valuable information about scar and fibrosis, they provide little information about biological processes that can critically contribute to the outcome of patients post-MI. Gadofosveset is an albumin-binding blood pool contrast agent that has been used to prolong the acquisition window to enable steady state high resolution angiography and to improve the delineation of the vasculature [52]. Experimentally, it has also been used in patients with carotid [39] and coronary [53] atherosclerosis for imaging the vessel wall. Gadofosveset is characterized by its significantly higher relaxivity when bound to albumin (r1 = 18-20 mmol^−1^s^−1^ at 1.5 T) [[36], [37], [38]]. The r1 relaxivity of unbound gadofosveset in PBS is r1_free_ ≈6.5 mmol/l compared to 5 mmol/L for Gd-DTPA and gadobutrol due to its higher molecular weight. The free fraction (unbound) was measured by simply diluting gadofosveset in PBS in the absence of albumin in solution [[36], [37], [38]]. When gadofosveset was incubated with murine plasma 60% binds to plasma albumin and 40% remains as a free fraction. Thus *in vivo*, both free and bound fractions of gadofosveset are present and both can leak through the disrupted endothelium of the coronary arteries or enter the myocardium through leaky neovessels. However, to which percentage the free and the found fraction enter the myocardium remains unknown. We suggest that three possible mechanisms may lead to gadofosveset uptake in the myocardium: [1] unbound gadofosveset leaks into the myocardium; [2] unbound gadofosveset leaks into the myocardium and binds to intra-myocardial albumin; and [3] bound gadofosveset leaks into the myocardium. Gadofosveset also has longer blood half life time due to its interaction with albumin allowing for a longer imaging window and enabling acquisitions during the steady-state. In the present study, we demonstrate that imaging with gadofosveset provides similar LGE areas as compared to Gd-DTPA but with higher signal intensity which facilitates scar visualization and delineation. At high field (7 T), the relaxivity of Gd-DTPA and gadofosveset are significantly lower compared with that at lower magnetic field strength (3.48 mmol^−1^s^−1^ and 4.56 mmol^−1^s^−1^, respectively) [54,55]. At lower, and clinically relevant, magnetic field strength gadofosveset would have even higher relaxivities and differentiation between the albumin-bound and free fraction of the agent would be possible using T1 mapping. This effect may further aid detection and analysis of the ischemic area.

After MI, the ischemic tissue undergoes severe changes in cellular and extracellular matrix composition leading to an increase in the extracellular volume [56,57]. Acute myocardial injury, defined by cardiomyocyte death and endothelial damage, leads to increased leakage of plasma macromolecules [58]. Gadofosveset has been recently used to assess focal changes in vascular permeability and remodeling in animal models of atherosclerosis and patients with cardiovascular disease, as its extravasation occurs mainly though damaged endothelium or microvessels [[39], [40], [41], [42], [43]]. In this study, the highest permeability occurred 3 days post-MI which was reflected by a significant increase in the R_1_ values in the infarct after injection of gadofosveset. This is most likely due to the acute damage of the cardiac endothelium, which allows non-restricted leakage of macromolecules, such as albumin, from the blood into the ventricular wall, leading to a higher permeability that it is not associated with ventricular remodeling. This result is in good agreement with the *ex vivo* histological validation where highest and diffused albumin extravasation is present 3 days post-MI. At later time points (10 and 21 days post-MI), increased R_1_ values after gadofosveset administration where observed in areas of the LV wall with increased microvessel formation. In contrast, R_1_ values measured after Gd-DTPA administration did not change between 3 and 21 days, thus providing limited information about LV remodeling post-MI. Moreover, both Gd-DTPA and gadofosveset led to higher R_1_ values in infarcted tissue compared to remote myocardium, demonstrating that both contrast agents preferentially extravasate into injured tissues. Similarly to our results, Saed et al. demonstrated the presence and distribution of abnormal microvascular hyperpermeability in a rat model of ischemia-reperfusion using a different albumin-binding MR contrast agent (albumin-(biotin)10-(Gd-DTPA)25) [30].

Gd-DTPA and gadofosveset have been also compared in other animal models and man. Using a canine model of ischemia reperfusion, Gd-DTPA showed better characteristics for myocardial scar visualization compared with Gd-BOPTA (MultiHance) and gadofosveset. However, a single imaging session 90min after reperfusion of the hearts was performed in these studies and a thus the temporal changes of contrast uptake within the remodeled myocardium was not investigated [59]. In patients with chronic MI, the number of segments and the transmurality of scar were underestimated by gadofosveset as compared to Gd-DTPA [35]. However, the accuracy of LGE images with gadofosveset was higher compared with those reported for Gd-DTPA. Additionally, in these studies only LGE MRI was used and no T1 mapping quantification was performed. In our study, we implemented LGE and also T1 mapping protocols that allowed quantification of not only the LGE area but also myocardial relaxation rate that allows for more quantitative assessment of contrast agent uptake.

The noninvasive assessment of cardiac permeability and neovascularization in the infarcted myocardium may be a useful tool to characterize ventricular remodeling after MI and may lead to a more accurate diagnosis and treatment guidance by differentiating the acute for the maturation phase of the myocardial remodeling process. This study demonstrates that gadofosveset has the potential to visualize and quantify microvascular changes associated with post infarction healing, allowing the differentiation between the acute and maturation phases post-MI. This approach could provide new valuable information for the stratification of patients in order to use a more targeted therapeutic intervention.

### 4.1 Limitations

One of the limitations of our study is the high dose of contrast agent used (10 fold compared to the clinically approved dose) due to low signal intensity obtained at the 7 T pre-clinical scanner and probably because of the use of intraperitoneal injection as opposed to the clinical standard intravenous injection, also resulting in a prolonged washout period. Therefore, additional studies are needed to investigate the optimal contrast agent dose administered at lower field strength (e.g. 3 T). In addition, to obtain better imaging quality of the ischemic myocardium, a wash out period of gadofosveset from blood is required, which would delay the imaging protocol. An additional limitation is the use of a permanent ligation animal model of MI. Additional studies using an ischemia-reperfusion model could potentially increase the clinical applicability of this study. In addition, because of the limited number of animals used in this study we did not have enough specimens to quantify the increase in albumin/CD31 staining by complimentary *ex vivo* methods. Our aim was to provide a proof of principle on the potential mechanism underlying the uptake of contrast agent into the myocardium but not to perform an extensive *ex vivo* quantification of the changes in albumin or neovessels density.

### 4.2 Conclusion

We demonstrate that both Gd-DTPA and gadofosveset provide comparable measurements of ischemic area. Additionally, the combination of LGE and T1 mapping after administration of gadofosveset allowed for the detection of changes in myocardial permeability differentiating acute and chronic phases following MI.

## Conflicts of interest

The authors declared they do not have anything to disclose regarding conflict of interest with respect to this manuscript.

## Financial support

This work was supported by the (1) British Heart Foundation Centre of Excellence at King's College London, (2) a British Heart Foundation Program grant (RG/12/1/29262), (3) the Wellcome EPSRC Centre for Medical Engineering at King's College London (WT, 203148/Z/16/Z) and (4) the Department of Health through the National Institute for Health Research (NIHR) comprehensive Biomedical Research Centre award to Guy's & St Thomas' NHS Foundation Trust in partnership with King's College London and King's College Hospital NHS Foundation Trust.

## Author contributions

Author contributions: B.L. and A.P. conception and design of research; B.L., A.P. and X.D. performed experiments; B.L., A.P. and S.L. analyzed data; B.L. A.P., S.L., A.Ph., R.B., and A.M.S. interpreted results of experiments; B.L. prepared figures; B.L. and A.P. drafted manuscript; B.L., A.P., A.Ph., R.B., and A.M.S. edited and revised manuscript; B.L., A.P., A.Ph., R.B., and A.M.S. approved final version of manuscript.
